# Evidence That the Etiology of Congenital Hypopituitarism Has a Major Genetic Component but Is Infrequently Monogenic

**DOI:** 10.3389/fgene.2021.697549

**Published:** 2021-08-11

**Authors:** Youn Hee Jee, Mariam Gangat, Olga Yeliosof, Adrian G. Temnycky, Selena Vanapruks, Philip Whalen, Evgenia Gourgari, Cortney Bleach, Christine H. Yu, Ian Marshall, Jack A. Yanovski, Kathleen Link, Svetlana Ten, Jeffrey Baron, Sally Radovick

**Affiliations:** ^1^Eunice Kennedy Shriver National Institute of Child Health and Human Development, National Institutes of Health, Bethesda, MD, United States; ^2^Division of Pediatric Endocrinology Rutgers Robert Wood Johnson Medical School Child Health Institute of New Jersey, New Brunswick, NJ, United States; ^3^Division of Pediatric Endocrinology, MedStar Georgetown University Hospital, Washington, DC, United States; ^4^Division of Pediatric Endocrinology, Walter Reed National Military Medical Center, Bethesda, MD, United States; ^5^Section of Adult and Pediatric Endocrinology and Metabolism, University of Chicago, Chicago, IL, United States; ^6^Division of Pediatric Endocrinology, Pediatric Subspecialists of Virginia, Fairfax, VA, United States; ^7^Pediatric Endocrinology, Richmond University Medical Center, Staten Island, NY, United States

**Keywords:** congenital hypopituitarism, monogenic, digenic, ectopic posterior pituitary gland, combined pituitary hormone deficiencies

## Abstract

**Purpose:**

Congenital hypopituitarism usually occurs sporadically. In most patients, the etiology remains unknown.

**Methods:**

We studied 13 children with sporadic congenital hypopituitarism. Children with non-endocrine, non-familial idiopathic short stature (NFSS) (*n* = 19) served as a control group. Exome sequencing was performed in probands and both unaffected parents. A burden testing approach was used to compare the number of candidate variants in the two groups.

**Results:**

First, we assessed the frequency of rare, predicted-pathogenic variants in 42 genes previously reported to be associated with pituitary gland development. The average number of variants per individual was greater in probands with congenital hypopituitarism than those with NFSS (1.1 vs. 0.21, mean variants/proband, *P* = 0.03). The number of probands with at least 1 variant in a pituitary-associated gene was greater in congenital hypopituitarism than in NFSS (62% vs. 21%, *P* = 0.03). Second, we assessed the frequency of rare, predicted-pathogenic variants in the exome (to capture undiscovered causes) that were inherited in a fashion that could explain the sporadic occurrence of the proband’s condition with a monogenic etiology (*de novo* mutation, autosomal recessive, or X-linked recessive) with complete penetrance. There were fewer monogenic candidates in the probands with congenital hypopituitarism than those with NFSS (1.3 vs. 2.5 candidate variants/proband, *P* = 0.024). We did not find any candidate variants (0 of 13 probands) in genes previously reported to explain the phenotype in congenital hypopituitarism, unlike NFSS (8 of 19 probands, *P* = 0.01).

**Conclusion:**

Our findings provide evidence that the etiology of sporadic congenital hypopituitarism has a major genetic component but may be infrequently monogenic with full penetrance, suggesting a more complex etiology.

## Introduction

The embryonic development of the pituitary gland is a complex process involving formation of Rathke’s pouch, interactions with the developing ventral diencephalon, and differentiation of multiple pituitary cell types (Bancalari et al., 2012). These structural and cellular developmental processes are orchestrated by complex molecular mechanisms involving the interplay of multiple signaling pathways and transcription factors such as *HESX1, SOX2/3, TBX2/3, LHX2/3/4, SIX3/6*, *PITX1/PITX2, PROP1, POU1F1*, and *TPIT* (Rizzoti, 2015). Abnormalities in this developmental program can result in congenital hypopituitarism, a disorder characterized by presence of one or more pituitary hormone deficiencies. Often these functional pituitary deficits are accompanied by structural pituitary abnormalities, such as a hypoplastic or absent anterior pituitary gland and/or an ectopic posterior pituitary gland.

Although much has been learned about pituitary development from mouse models, the etiology can only be identified in a minority of patients with congenital hypopituitarism. In some affected patients, monogenic etiologies have been identified, including genetic defects that are often recessive in *HESX1* (Thomas et al., 2001), *PROP1* (Wu et al., 1998; Correa et al., 2019), *POU1F1* (Turton et al., 2005; Birla et al., 2019), *LHX3* (Rajab et al., 2008; Jullien et al., 2019), and *LHX4* (Tajima et al., 2007). However, a monogenic etiology can be identified in fewer than 10% of cases (Castinetti et al., 2015); in the remainder, the etiology is generally unclear. These idiopathic congenital cases are usually sporadic; familial cases of isolated congenital hypopituitarism are less commonly observed (Zwaveling-Soonawala et al., 2018), suggesting that single-gene defects may not be the dominant cause. In some patients, pathogenic variants are suspected but are also found in unaffected relatives, suggesting incomplete penetrance due to additional unknown contributing factors (Bashamboo et al., 2016, 2017; Cohen et al., 2017; Hovinga et al., 2018; Babu et al., 2019; Dateki et al., 2019). These observations suggest that the etiology of congenital hypopituitarism is often more complex than a simple monogenic defect. A possible explanation is that, as has been described for idiopathic hypogonadotropic hypogonadism congenital hypopituitarism may often have a digenic or oligogenic etiology. Indeed, cases of digenic inheritance have been reported (McCormack et al., 2017). A second possibility is that there are major non-genetic causative factors. For example, an association between congenital hypopituitarism and adverse perinatal events with breech presentation has been observed (Maghnie et al., 1991), suggesting an etiological role for birth asphyxia (Kikuchi et al., 1988).

In this study, we asked two questions regarding the etiology of congenital hypopituitarism. First, in patients with sporadic congenital hypopituitarism, is there a major genetic component to the etiology? Second, in these patients, are monogenic causes common, involving as yet undiscovered genes? To address the first question—whether there is a major genetic component underlying sporadic congenital hypopituitarism—we studied a group of patients with non-familial congenital hypopituitarism and used exome sequencing to determine the frequency of rare, predicted-pathogenic variants in 42 genes that have been implicated in pituitary gland development (Zwaveling-Soonawala et al., 2018). As a control group, we used patients with non-familial short stature (NFSS) of unknown etiology who had been studied in parallel with the hypopituitary subjects, using the same methods for exome sequencing, data processing, and sequence analysis. These control subjects had been evaluated to exclude pituitary disease and thus would be expected to have a frequency of pituitary-related variants similar to that of the normal population.

To address the second question—whether sporadic congenital hypopituitarism often has a monogenic cause, involving as yet undiscovered genes—we studied the same patient population. However, in this second analysis, we searched for variants in any gene (not just those known to be involved in pituitary development) that was inherited in a pattern that would explain the apparent sporadic occurrence. Thus, we counted only rare, predicted-pathogenic variants that showed a recessive inheritance (homozygous or compound heterozygous in the proband with one variant inherited from each parent), an X-linked recessive inheritance (male probands only), or arose *de novo* (not present in either parent). We compared the frequency of such candidate variants in subjects with congenital hypopituitarism to the frequency in subjects with NFSS. In this analysis, NFSS subjects allowed us to compare the relative frequency of potential monogenic causes in the two conditions.

## Materials and Methods

### Subjects and Sample Collection

Thirteen patients with congenital hypopituitarism (age 4–31 years, 5 males) and their unaffected biological parents were studied. Based on MRI evaluation, 10 probands had hypoplastic or absent anterior pituitary glands with ectopic posterior pituitaries and 3 probands had hypoplastic or absent anterior pituitary gland and absence of the bright spot that corresponds to the posterior pituitary gland. Affected subjects had either isolated GH deficiency (*n* = 1) or combined with other pituitary hormone deficiencies (*n* = 12; Table 1). Growth hormone deficiency was diagnosed with either provocative testing using 2 different stimuli or low IGF-1 in the context of other pituitary hormone deficiencies and pituitary abnormalities on MRI. No affected subjects had a history of consanguinity or a family history of pituitary disease. No patients had a history of adverse perinatal events including birth asphyxia or breech delivery. TSH deficiency was diagnosed based on a low serum free T4 with serum TSH < 10 mIU. ACTH deficiency was diagnosed with ACTH stimulation based on a peak serum cortisol < 18 μg/dL after receiving 250 micrograms of ACTH_1__–__2__4_ intravenously. FSH/LH deficiency was diagnosed when subjects failed to enter puberty. One subject had diabetes insipidus.

**TABLE 1:** Characteristics of subjects with congenital hypopituitarism.

Age^†^	Pituitary hormone deficiencies	Anterior pituitary gland on MRI	Posterior pituitary on MRI
10–15	GH, partial ACTH, TSH	HAP	EP
16–20	GH, ACTH, TSH, LH/FSH	HAP	EP
16–20	GH, ACTH, TSH, LH/FSH	AAP	EP
6–10	GH	HAP	EP
20–25	GH, ACTH, TSH, LH/FSH	AAP	EP
15–20	GH, ACTH, TSH, LH/FSH	HAP	X
20–25	GH, ACTH, TSH, LH/FSH	HAP	EP
16–20	GH, ACTH, TSH, LH/FSH	AAP	X
30–31	GH, ACTH, TSH, LH/FSH	HAP	EP
0–5	GH, ACTH, TSH	HAP	EP
15–20	GH, ACTH, TSH, LH/FSH	AAP	EP
6–10	GH, ACTH, TSH, LH/FSH	HAP	X
0–5	GH, ACTH, TSH	HAP	EP

*HAP, hypoplastic anterior pituitary gland; AAP, aplastic anterior pituitary gland; EP, ectopic posterior pituitary gland; X, Not visualized on pituitary MRI. ^†^Age (y) range was provided per journal policy and sex of individual subjects was omitted to protect privacy per journal policy.*

Nineteen subjects with NFSS and their unaffected biological parents were also studied. None of these subjects (age 3–42 years, male = 12) had a history of pituitary hormone deficiencies. On clinical and biochemical evaluation, they had no evidence of central hypothyroidism. Similarly, all NFSS subjects either passed growth hormone provocative testing or showed no evidence of growth hormone deficiency to justify provocative testing. There was no history of consanguinity. All affected subjects’ parents had normal heights suggesting monogenic inheritance (Table 2). At the time of presentation, the affected subjects had no clinical findings revealing a commonly known genetic cause of diminished linear growth evident either to their health care providers or to the study investigators. The characteristics of these subjects are provided in detail in Table 2. Nine patients had isolated short stature whereas 10 patients had other accompanying abnormal features.

**TABLE 2:** Characteristics of subjects with non-familial short stature.

Age^†^	Height SDS at diagnosis	PH/MPH^¶^ (SDS)	Associated dysmorphic features
40–45	**−2.1**	**−**0.5/**−**0.1**/−0.3**	Disproportionate short stature, Noonan-like facies
6–10	**−2.1**	**−**1.1/0.1**/−0.6**	None
6–10	**−4.63**	**−**0.3/0.5**/0.1**	Multiple dysmorphic features
6–10	**−2.9**	0.1/**−**0.7**/−0.2**	None
35–40	**−2**	**−**1.2/0.4**/−0.4**	None
5–10	**−2.4**	0.5/**−**0.1**/0.7**	Speech delay
35–40	**−1.9**	**−**0.2/0.1**/0.1**	None
15–20	**−2.1**	1.9/0.4**/1.1**	Disproportionate short stature
5–10	**−3.8**	0.5//1.1**/0.7**	Joint laxity, muscular build
5–10	**−2.6**	0.5/**−**0.5**/0.00**	Disproportionate short stature, facial dysmorphism, Chiari I malformation, developmental delay, Shawl scrotum, developmental delay
10–15	**−1.9** (on GH)	0.1/**−**1.4**/−0.6**	Early puberty
5–10	**−2.7**	**−**0.8/**−**1.1**/−0.9**	None
0–5	**−4.6**	0.5/**−**0.1**/0.3**	None
6–10	**−1.75**	**−**0.76/0.7**/−0.1**	Midface hypoplasia Disproportionate short stature
6	**−2.6**	**−**0.2/0.7/**0.1**	Dysmorphic facial features
8	**−2**	**−**0.6/**−**0.3/**−0.5**	None
8	**−2**	**−**0.6/0.3/**−0.1**	None
8	**−2.2**	0.9/**−**0.3/**0.3**	None
3	**−5.07**	**−**2/**−**2.1**/−2**	Developmental delay, dysmorphic facial features

*^¶^PH/MPH: Father’s height/mother’s height/Mid-parental height. ^†^Age (y) range was provided per journal policy and sex of individual subjects was omitted to protect privacy per journal policy.*

Genomic DNA from white blood cell in all patients was extracted for genetic testing. The study (clinicaltrials.gov/ct2/show/NCT02311322) was approved by the NICHD IRB. All adult subjects and parents of minors provided written informed consent and children provided written assent.

### SNP Array and Exome Sequencing and Data Analysis

#### SNP Array and Exome Sequencing

SNP array was performed as previously described (Jee et al., 2017). SNP array data were analyzed for copy-number variations (CNVs) previously reported to be associated with congenital hypopituitarism (Correa et al., 2018), as well as to confirm paternity and rule out parental consanguinity.

Exome sequencing was performed on probands and the parents as trios at the National Institutes of Health Sequencing Center. The detailed method was previously published (Jee et al., 2017). In brief, Illumina sequencing libraries were generated from 100 ng genomic DNA using the Accel-NGS 2S DNA Library Kit (Swift Biosciences) on a Beckman Coulter Biomek FX robot. The median insert size was approximately 350 bp. Libraries were tagged with unique dual index DNA barcodes to allow pooling of libraries and minimize the impact of barcode hopping. Libraries were pooled in groups of 8 for exome enrichment using the xGen Exome Research Panel v1.0 (IDT). Multiple enriched pools were combined for sequencing on the NovaSeq 6000 (Illumina) to obtain at least 35 million 150-base read pairs per individual library. Raw data were processed using RTA version 3.4.4 for base calling. All subjects sequencing data were processed in parallel for alignment and variant calling. The combined data were formatted in a single .vs file file and analyzed using VarSifter (Teer et al., 2012) which enables a search for variants in a specific gene and analysis of genotypes using Boolean logic.

##### Data Analysis 1: Frequency of Candidate Variants in Genes Associated With Pituitary Development

We first identified variants in 42 genes (Supplementary Table 1) previously reported to be associated with pituitary development, including 40 genes compiled by Zwaveling-Soonawala et al. (2018) and 2 additional genes that were found associated with pituitary hormone deficiencies in humans. These 42 genes include: (1) genes previously reported in patients with congenital hypopituitarism, (2) genes for which a genetic mouse model showed pituitary abnormalities, or (3) genes encoding proteins that could interact with signaling pathways important for pituitary gland development. Variants were considered potentially pathogenic candidates if they met all of the following criteria: (1) sequencing coverage > 10 reads and most probable genotype (MPG)/coverage > 0.5 (Adams et al., 2012), (2) confirmed to be present by visual inspection of Binary Alignment Map (BAM) files, (3) population frequency < 1% in the Genome Aggregation Database (gnomAD) v2.1.1 data set (GRCh37hg19), and homozygous variants found in < 2 subjects in gnomAD (which includes approximately 150,000 subjects), and (4) altered the predicted amino acid sequence of the encoded protein (i.e., missense, non-sense, frameshift/non-frameshift insertions or deletions, and splicing variants), and (5) predicted to be pathogenic by at least 2 out of 3 prediction algorithms (SIFT, MutationTaster, PolyPhen2) (Adzhubei et al., 2010; Schwarz et al., 2014; Vaser et al., 2016). CADD score was not included as one of the prediction criteria but is provided as Supplementary Information. For this analysis, variants that met the above criteria were sought in probands regardless of inheritance pattern in order to broadly screen for any genetic contribution, including incomplete penetrance and di/oligogenic causes (Supplementary Figure 1). This analysis was performed separately in subjects with congenital hypopituitarism and in subjects with NFSS, and the frequency of rare, predicted-pathogenic variants was compared in the two groups. The latter group, which had no evidence of pituitary disease, served as a control group for this burden testing approach.

##### Data Analysis 2: Frequency of Candidate Variants for a Monogenic Etiology

We next sought genetic variants in any protein-coding gene in the genome, not just genes associated with pituitary development. We used the same 5 criteria used in Data Analysis 1 to identify rare, predicted-pathogenic variants. We next narrowed the list to include only variants that were inherited in a pattern consistent with a monogenic etiology in these families that had an affected proband and two unaffected parents. These patterns included: (1) an autosomal recessive inheritance in which the proband had a homozygous or compound heterozygous variant in a gene with one allele inherited from each parent; (2) X-linked recessive inheritance in which a male proband inherited the variant from his mother; or (3) *de novo* occurrence in which a heterozygous variant was found in the proband but was absent in both parents (Supplementary Figure 1). This analysis was performed separately in subjects with congenital hypopituitarism and in subjects with NFSS, and the frequency of rare, predicted-pathogenic variants was compared in the two groups to compare the frequency of potential monogenic inheritance in the two conditions.

### Statistical Analysis

Statistical analyses were performed using SPSS (v25, IBM, Armonk, NY). The average number of variants per proband was compared in the two groups (congenital hypopituitarism vs. NFSS) using the Mann-Whitney *U*-test. The percent of probands with at least 1 variant was compared in the two groups with the Fisher exact test.

## Results

### SNP Array Analyses

SNP array analyses confirmed paternity and non-consanguinity in all families and excluded the presence of significant copy-number variations.

### Rare, Predicted-Pathogenic Variants in Genes Associated With Pituitary Gland Development Are Enriched in Subjects With Congenital Hypopituitarism

**FIGURE 1:**
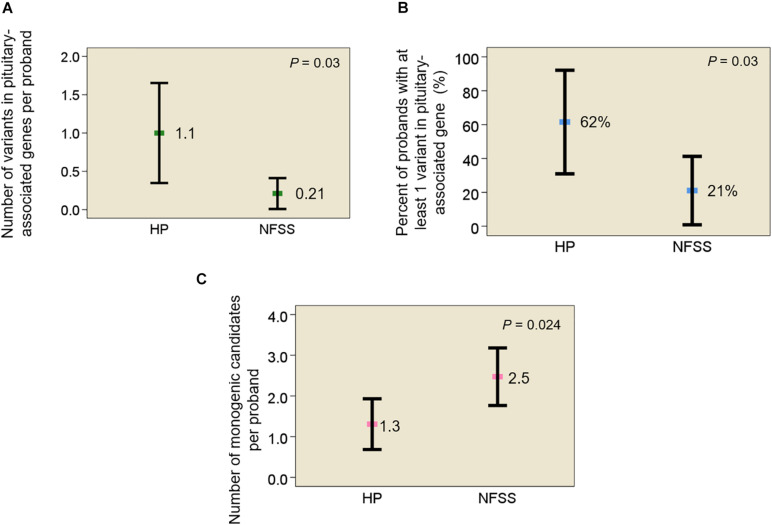
Rare, predicted-pathogenic variants in subjects with congenital hypopituitarism and non-familial short stature. **(A)** Mean number of rare, predicted-pathogenic variants per proband in 42 genes associated with pituitary development. **(B)** Percent of probands with at least 1 variant in any of 42 pituitary-associated genes. **(C)** Mean number of monogenic candidates for the condition (hypopituitarism or non-familial short stature) per proband. Square indicates mean. Error bar shows 95% confidence interval. HP, congenital hypopituitarism; NFSS, non-familial short stature.

In data analysis 1, using exome sequencing, we searched for rare, predicted-pathogenic sequence variants in 42 genes associated with pituitary gland development. The average number of these variants per proband was greater in subjects with congenital hypopituitarism (*n* = 13 probands) than in in the control subjects (*n* = 19 probands) with NFSS (1.1 vs. 0.21 mean variants per proband, *P* = 0.03, Figure 1A). Similarly, the percent of probands with at least 1 variant in any of the 42 pituitary-associated gene was greater in subjects with congenital hypopituitarism than in subjects with NFSS (62% vs. 21%, *P* = 0.03, Figure 1B). Rare, predicted-pathogenic variants were found in 11 genes (*ARID1B, CDON, CHD7, GLI1, GLI4, LHX3, LHX4, SIX1, SIX5, SIX6, SOX3*) in 8 subjects with congenital hypopituitarism (62%) and in 3 genes (*CHD7, LHX4*, and *WNT5A*) in 4 subjects with NFSS (21%) (Table 3 and Supplementary Table 1). Two subjects with congenital hypopituitarism carried more than one rare, predicted-pathogenic variant: one subject with congenital hypopituitarism carried variants in *LHX4* and *CDON* inherited from her father and a variant in *SIX5* from her mother; the other subject with congenital hypopituitarism carried an in-frame variant in the polyalanine region of *SOX3* from her father and variants in *GLI1* and *CHD7* (Gregory, 2020) from her mother. Hughes et al. (2013) showed that changes in the polyalanine expansion could cause a partial loss-of-function in the protein and Alatzoglou et al. (2011) reported a patient with hypopituitarism due to deletion in the polyalanine tract (Alatzoglou et al., 2011; Hughes et al., 2013). However, because the subject’s father, who had no evidence of hypopituitarism, also carried it, this variant alone does not appear sufficient on its own to cause hypopituitarism; however, it remains possible that this variant could contribute to abnormal development of the pituitary gland in concert with other genetic variants. None of the variants found in subjects with congenital hypopituitarism were inherited in a pattern that could explain the sporadic presentation with a fully penetrant autosomal recessive, X-linked recessive, or *de novo* inheritance. Instead, we observed primarily heterozygous variants inherited from an unaffected parent, suggesting that these variants, if pathogenic, show incomplete penetrance, possibly due to a digenic or oligogenic etiology or to interacting non-genetic factors, as has been proposed (Hong and Krauss, 2012). There was no specific phenotype correlated with the identified variants in these patients.

**TABLE 3:** Genetic variants found in 42 known pituitary-associated genes.

Gene (variant, hg19)	Variant type	Inheritance from	Population frequency in gnomAD	Evidence for a role in pituitary gland
**Congenital hypopituitarism**				
*CDON* (chr11: 125887183 C > T) *ARID1B* (chr6: 157454234 C > T)	Missense Missense	Mother Mother	Not found 0.004	Reported to be associated with pituitary stalk interruption (Bashamboo et al., 2016) Reported to be associated with growth hormone deficiency in human and mice (Yu et al., 2015; Celen et al., 2017)
*SIX6* (chr14: 60976501 G > A)	Missense	Mother	0.007	Expressed in pituitary gland during development and interacts with PITX1 and PITX2 (Jean et al., 1999; Xie et al., 2015)
*SIX1* (chr14: 61113110 G > A)	Missense	Mother	0.003	Reported to induce pituitary placode (Zimmer et al., 2016)
*LHX4* (chr1: 180235637 G > A) *CDON* (chr11: 125859642 C > T) *SIX5* (chr19: 46268833 C > T)	Missense Non-sense Missense	Father Father Mother	Not found 0.00006 0.0002	Reported in patients with CH (Cohen et al., 2017) Reported to be associated with pituitary stalk interruption (Bashamboo et al., 2016) Member of SIX family and high expression in mouse and human pituitary gland (http://biogps.org/#goto)
*ARID1B* (chr6: 157522148 C > A)	Missense	Mother	0.00001	Reported to be associated with growth hormone deficiency in human and mice (Yu et al., 2015; Celen et al., 2017)
*GLI1* (chr12: 57865830-AA-A) *SOX3* (chrX: 139586486-CAGCGGCGGCGGCCG CGGCAGC-C) *CHD7* (chr8:61693942_61693947 insGCAAAA)	frameshift Non-frameshift substitution Non-frameshift Insertion	Mother Father Mother	0.000006 Not found Not found	Involved in pituitary gland development in zebrafish (Wang et al., 2010) Reported in patients with CH (Alatzoglou et al., 2011) Reported in patients with CHARGE syndrome and CH (Gregory et al., 2013)
*GLI4* (chr8: 144358408 G > C)	Missense	Father	0.0001	Mediates Sonic Hedgehog signaling (Villavicencio et al., 2000)
*LHX3* (chr9: 139091601 G > T)	Missense	Father	0.0002	Reported in patients with CH (Jullien et al., 2019)
**Non-familial short stature**				
*CHD7 (chr*8: *61774876 A* > *G)*	Missense	Father	0.00001	Reported in patients with CHARGE syndrome and CH (Gregory et al., 2013)
*LHX4* (chr1: 180243593 C > T)	Missense	Mother	0.0002	Reported in patients with CH (Cohen et al., 2017)
*LHX4* (chr1: 180217489 A > G)	Missense	Mother	0.0001	Reported in patients with CH (Cohen et al., 2017)
*WNT5A* (chr3: 55508427 T > C)	Missense	Mother	0.00001	Involved in mouse pituitary gland development (Cha et al., 2004)

*CH, congenital hypopituitarism.*

### Candidate Variants That Could Account for a Fully Penetrant Monogenic Etiology Were Infrequently Found in Subjects With Congenital Hypopituitarism

In data analysis 2, we searched the exome sequencing data for variants in any gene (not just those known to be involved in pituitary development) in order to capture undiscovered genetic causes that were inherited in a fashion that could explain the sporadic occurrence of the proband’s condition (either hypopituitarism or short stature) with a monogenic, fully penetrant etiology. Thus, we counted only rare, predicted-pathogenic variants that showed a potentially recessive inheritance (homozygous or compound heterozygous in the proband with one allele inherited from each parent), an X-linked recessive inheritance (male probands only), or arose *de novo* (not present in either parent) because these inheritance patterns could explain the presence of the condition in the proband and the absence in the parents. Interestingly, there were fewer of these monogenic candidates in subjects with congenital hypopituitarism than in the subjects with NFSS (1.3 vs. 2.5 candidates per proband, *P* = 0.024, Figure 1C and Table 4). Furthermore, none of these variants (Table 4) were found in genes that have been reported to cause pituitary hormone deficiencies or anatomic brain abnormalities and indeed variants in many of these genes are associated with disorders that do not involve the pituitary (Table 4), suggesting that most of these variants are not responsible for the hypopituitarism. In contrast, in subjects with NFSS, we found variants in multiple genes that have been found to cause monogenic short stature, including *BRF1* (Cerebellofaciodental Syndrome) (Jee et al., 2017), *QRICH1* (Ververi-Brady Syndrome) (Lui et al., 2019), *FBN1* (acromelic dysplasia), *HUWE1* (X-linked mental retardation syndrome*), SRCAP* (Floating-Harbor syndrome), *ACAN* (short stature and advanced bone age) (Tatsi et al., 2017), *ZEB* (Mowat-Wilson Syndrome), *and CUL7* (3-M syndrome). The causality of the variants found in these genes was supported by bioinformatic analysis and either a highly specific clinical phenotype consistent with the known disorder or a consistent but less specific phenotype combined with functional studies. Thus, candidate variants in genes previously reported to explain the phenotype were identified in 0 of the 13 trios with congenital hypopituitarism and in 8 of 19 trios (42%) with NFSS (*P* = 0.01; Table 2). These findings suggest that a monogenic inheritance is substantially less common in sporadic congenital hypopituitarism than in sporadic short stature.

**TABLE 4:** Candidate monogenic variants.

**Congenital hypopituitarism**			

Inheritance mode	Gene	Known associated disease	Gene function/tissue expression/mouse model
*De novo* AR (compound het) XLR	*TTLL4 TNXB* .	. Ehlers-Danlos syndrome (No CE) .	*TTLL4*: Required for cytoskeletal organization (Ijaz et al., 2017)/NS/NA *TNXB*: extracellular matrix glycoproteins/NS/abnormal skin tensile strength

*De novo* AR XLR	*KLHDC4* . .	. . .	*KLHDC4*: Orphan member of the kelch repeat superfamily, possibly involved in cell proliferation and migration (Lian et al., 2016)/NS/NA

*De novo* AR (compound het) XLR	*MARCH3 PER2 FLG* .	. Familial advanced sleep phase syndrome (No CE) Ichthyosis Vulgaris (No CE)	*MARCH3*: E3 ubiquitin ligase (Fatehchand et al., 2016)/NS/available but pituitary phenotype was not investigated (Lin et al., 2018) *PER2*: circadian pace maker/high expression in rat pituitary gland/disrupted circardian rhythm and cancer prone (Fu et al., 2002) *FLG*: intermediate filament-associated protein/NS/percutaneous allergen priming (Fallon et al., 2009)

*De novo* AR (compound het) XLR	*CRHR1* . *GPC3*	. . Type 1 Simpson-Golabi-Behmel syndrome (No CE)	*CRHR1*: CRH receptor/high expression in human pituitary gland in human/*Crh1*(-/-) mice show upregulated Acth receptor in pituitary and adrenal gland (Müller et al., 2001) *GPC3*: control of cell division and growth regulation/NS/controls limb patterning and skeletal development (Paine-Saunders et al., 2000)

*De novo* AR (homozygous)	. *TTLL6 DCAKD*	. .	*TTLL6*: polyglutamylase enzyme and regulates cilia structure and motility (Lee et al., 2012)/NS/NA *DCAKD*: Parkinson disease high risk loci (Barbu et al., 2020)/NS/NA

*De novo* AR	. .	. .	. .

*De novo* AR	. .	. .	. .

*De novo* Compound het	. *PDE4DIP*	. .	. *PDE4DIP*: phosphohydrolases, involved in signal transduction and hydrolyze 3’ cyclic phosphate bonds in 3’, 5’-cGMP and 3’,5’-cAMP (Shapshak, 2012)/NS/NA

*De novo* AR (compound het)	. *CEP128*	. .	*CEP128*: regulates TGF-β/BMP singling at the primary cilium (Mönnich et al., 2018)/NS/NA

*De novo* AR (homozygous)	. *MET*	. Autosomal recessive deafness (No CE)	*MET*: receptor tyrosine kinase/NS/tumorigenesis (Graveel et al., 2009)

*De novo* AR (homozygous) X-linked	. *PRRG2* .	. .	*PRRG2*: serves as binding partner for multiple proteins (Yazicioglu et al., 2013)/NS/NA

*De novo* AR (homozygous) AR (compound het)	*GJB5 ACADVL RGPD3*	. Very long-chain acyl CoA dehydrogenase deficiency (No CE) .	*GJB5*: involved in trophoblast stem cell differentiation (Kibschull et al., 2014)/NS/available but pituitary phenotype was not investigated (Zheng-Fischhöfer et al., 2007) *ACADVL*: Catalytic enzyme/NS/hepatic steatosis (Cox et al., 2001) *RGPD3*: GTPase activator, reported to be associated with craniofacial morphology (Wu et al., 2019)/high expression in human pituitary gland/NA

*De novo* AR	. .	. .	. .

**Non-familial short stature**		

**Inheritance**	**Gene**	**Known associated disease or role in linear growth**

*De novo* AR (compound het)	**Potential new genetic cause** *SMARCD2 TMBIM1*	**Likely role in linear growth** Autosomal recessive neutropenia (No CE) No known association with linear growth

*De novo* AR (homozygous) (compound het)	. *APLP2 ZFHX3*	. No known association with linear growth No known association with linear growth

*De novo* AR (compound het) XLR	. ***BRF1* (causative variant)** *MCM3AP* .	. **Cerebellofaciodental syndrome (CE)** Autosomal recessive peripheral neuropathy (No CE) .
*De novo* AR (compound het)	. *SDSL*	. Congenital neutropenia (no CE)

*De novo* AR (compound het) XLR	**Potential new genetic cause***FZD6 HECW2 WDR52* .	**Likely role in linear growth** Autosomal receive Nail disorder (no CE) Neurodevelopmental disorder (no CE) Autosomal recessive spermatic failure (no CE) .

*De novo* AR	***QRICH1* (causative variant)** *PQLC2*	**Ververi-Brady syndrome (CE)** No known association with linear growth

*De novo* AR (compound het)	*FBXO41 PEX6*	No known association with linear growth Zellweger syndrome (no CE)

*De novo* AR (compound het) XLR	*PDCD6IP***Potential new genetic cause***SRRM2* .	No known association with linear growth **Likely role in linear growth** No known association with linear growth .

*De novo* AR XLR	***FBN1* (causative variant)** . .	**Acromelic dysplasia (CE)** . .

*De novo* AR XLR	*ZNF506* . ***HUWE1* (causative variant)**	No known association with linear growth . **X-linked syndromic mental retardation, Turner type (CE)**

*De novo* AR XLR	***SRCAP* (causative variant)** . .	**Floating-Harbor syndrome (CE)** . .

*De novo* AR XLR	. *MMP8 SPTB*	. No known association with linear growth Type 2 spherocytosis (No CE)

*De novo* AR (homozygous) AR (compound het)	*DDX46 MRGPRX1 FTCD MCOLN3 RYR1 MLH1*	No known association with linear growth No known association with linear growth Autosomal recessive glutamate formiminotransferase deficiency (no CE) No known association with linear growth Neuromuscular disease (no CE) Autosomal recessive Mismatch mismatch repair cancer syndrome (no CE)

*De novo* AR XLR	***ACAN* (causative variant)** . *BCOR NAP1L2 ACTRT1 MAGEC2*	**Short stature with advanced bone age (CE)** . Syndromic microphthalmia (no CE) No known association with linear growth No known association with linear growth No known association with linear growth

*De novo* AR XLR	***ZEB2* (causative variant)** . *COL4A5*	**Mowat-Wilson syndrome (CE)** . X-linked Alport syndrome (no CE)

*De novo* AR XLR	. . .	. . .

*De novo* AR (compound het)	*CYFIP2 IL7R NRAP*	Epileptic encephalopathy (no CE) Severe combined immunodeficiency (no CE) No known association with linear growth

*De novo* AR XLR	**Potential new genetic cause***LRP1B* .	**Likely role in linear growth** No known association with linear growth .

*De novo* AR (homozygous) **AR (compound het)** XLR	. *PLBD1 HEG1* ***CUL7* (causative variant)** *APOOL*	. No known association with linear growth No known association with linear growth **3-M syndrome (CE)** No known association with linear growth

*AR, autosomal recessive; compound het, compound heterozygous; XLR, X-linked recessive; No CE, the subject had no clinical evidence for the disorder listed; CE, the subject had clinical evidence for the disorder listed. Causal genes and strong candidate genes are in bold; NS, non-specific expression (reference: http://biogps.org/#goto); NA, not available. Mouse phenotype was searched at http://www.informatics.jax.org.*

## Discussion

The first major finding of this study is that rare, predicted-pathogenic variants in genes associated with pituitary development were enriched in subjects with congenital hypopituitarism compared to a control group of subjects with NFSS. In the 13 subjects with congenital hypopituitarism in our study, rare variants that are predicted to be pathogenic were found in 10 genes implicated in pituitary development including 8 genes (*ARID1B, CDON, CHD7, LHX3, LHX4, PAX6, SIX6*, and *SOX3*) reported to be associated with pituitary hormone deficiencies in humans. Some of these variants may have been found incidentally and not be causative. However, only 0.26 such variants per proband were found in the control group, which suggests that only a few of the variants found in the subjects with hypopituitarism were incidental. Thus, the findings from this first burden analysis suggest that the etiology of sporadic congenital hypopituitarism has a major genetic component.

The second major finding from this study is that the number of monogenic candidate variants was lower in subjects with congenital hypopituitarism than in those with NFSS. For this analysis, we searched for rare, predicted-pathogenic variants that were inherited in a fashion (autosomal recessive, X-linked recessive, or *de novo* occurrence) that could explain the condition (either congenital hypopituitarism or NFSS) as a monogenic, fully penetrant disorder. This analysis was not restricted to genes involved in pituitary development but potentially included all protein-coding genes in the genome in order to capture undiscovered genetic causes. In the subjects with NFSS, we found an average of 2.5 monogenic candidates per proband including multiple genes previously reported to cause monogenic short stature, suggesting that NFSS frequently has a monogenic etiology. In contrast, in subjects with congenital hypopituitarism, we found fewer monogenic candidates, 1.3 per proband, and none of these had previously been shown to cause hypopituitarism or play an important role in pituitary or brain development. Taken together, the two analyses suggest that congenital hypopituitarism has a major genetic component but may have a more complex etiology than a simple monogenic disorder with complete penetrance. However, a large-scale study would be needed to confirm our findings. One possibility consistent with these observations is that congenital hypopituitarism often has a digenic or oligogenic inheritance, as has been suggested (Hamdi-Rozé et al., 2020), although data for this hypothesis has been reported only in a small number of families to date (Zwaveling-Soonawala et al., 2018). In our study, 2 out of 13 families showed a possible oligogenic etiology. Another possibility is that congenital hypopituitarism is often caused by a combination of an underlying genetic defect and a non-genetic insult. This possibility has been demonstrated in mice in which a combination of *Cdon* genetic ablation and prenatal exposure to ethanol impaired development of Rathke’s pouch (Hong and Krauss, 2012). A non-sense mutation in *CDON* (Bashamboo et al., 2016) was previously reported in a patient with pituitary stalk interruption syndrome, however the variant was also inherited from the unaffected parent, as we observed in one of our patients, suggesting the presence of other factor(s) contributing to the pathogenesis of the disease. Congenital hypopituitarism also has been associated with breech position and adverse perinatal events (Maghnie et al., 1991) suggesting a role for non-genetic factors; however, we did not observe the association in our cohort.

A strength of the study design is that it avoids several possible confounding factors that could affect the number of variants found in the two groups of subjects. Subjects in both groups were sequenced at a single sequencing center using the same methodology, and their data were aligned, genotyped, and variant-called in parallel. In addition, we sought to avoid false positive and negative variant calls by manually examining the BAM files for all observed rare variants with coverage > 10 sequencing reads and MPG/coverage ratio of > 0.5. However, this study did not have a control population of healthy subjects and parents who had undergone exome sequencing analysis using all the same methodologies as the subjects with hypopituitarism. An additional limitation is the small size of the study. Far more subjects would have been required to perform burden testing for individual genes, that is, to show an increased number of variants in one specific gene in the case-vs.*-*control analysis. For this reason, we instead performed burden testing on aggregate sets of genes—either genes associated with pituitary development or genes that were inherited in a pattern that matched the pedigree—thus greatly increasing the power to detect differences between the case and control groups. We chose not to compare variant frequencies with those in available public databases which would have introduced multiple confounding variables (Guo et al., 2018). These databases combine exome sequence data obtained using various sequencing platforms, and thus would not match the sequencing platform used for our subjects with hypopituitarism. Similarly, the sequence data would not have been jointly processed and variant-called with the samples from our hypopituitary samples, introducing additional potential systematic biases (Guo et al., 2018). In addition, coverage for each gene is not known, especially for the 42 genes that we examined in this study. Consequently, if these databases had been used, any observed differences in variant burden might be due to methodological differences between the case and control groups, rather than to real genetic differences. Furthermore, examining BAM files of available databases as a quality check on variant calls is not feasible with public databases. Therefore, instead, we compared data from patients with congenital hypopituitarism to a group of subjects with NFSS that had been evaluated for short stature and were not found to have evidence of pituitary disease and thus would be expected to have a frequency of pituitary-associated variants similar to the general population. Moreover, NFSS is often a monogenic disorder with full penetrance and thus serves as a useful comparator to congenital hypopituitarism for the analysis of monogenic candidates. A study design limitation is that exome sequencing data can identify only coding region variants or intronic variants that affect splicing, and thus misses non-coding regulatory variants, such as those in promoter regions, enhancers, or microRNAs. Further study exploring these regulatory regions may reveal additional important genetic cases of congenital hypopituitarism. An additional limitation is that the number of trios examined in our study may not be sufficient to represent the full spectrum of congenital hypopituitarism, which is likely to have a heterogenous etiology.

Our study findings are consistent with the findings of Zwaveling-Soonawala et al. (2018) who studied 20 patients with isolated pituitary stalk interruption syndrome and found multiple variants in genes associated with pituitary development or CNS development, which appeared to contribute to a polygenic etiology. McCormack et al. (2017) also reported the digenic inheritance of pathogenic variants in *PROKR2* and *WDR11* in a child with pituitary stalk interruption syndrome supporting the concept of a digenic cause for congenital hypopituitarism, and abnormal pituitary development. However, our study is novel in that it includes a control group that was sequenced and analyzed in parallel to allow rigorous comparisons of variant frequencies.

## Conclusion

In conclusion, we report that rare predicted-pathogenic variants in genes known to be associated with pituitary development are enriched in patients with sporadic congenital hypopituitarism, suggesting that the etiology of congenital hypopituitarism has an important genetic component. However, none of the variants found was inherited in a pattern that could explain the sporadic disorder with a fully penetrant monogenic etiology. Even when we widened our search beyond genes known to be associated with pituitary development, to capture undiscovered genes, we found few variants consistent with a fully penetrant monogenic etiology compared to subjects with NFSS. Thus, taken together, the findings suggest that genetic variants affecting pituitary development play a major role in the etiology of congenital hypopituitarism but that the disorder may not be a simple single-gene defect, at least in protein-coding regions.

## Data Availability Statement

The datasets presented in this study can be found online in dbGaP using accession number phs001617.v1.p1.

## Ethics Statement

The studies involving human participants were reviewed and approved by the National Institute of Child Health and Development. Written informed consent to participate in this study was provided by participants and the participants’ legal guardian/next of kin.

## Author Contributions

YJ, JB, and SR contributed to the conception and design of the study. YJ organized the database, performed the statistical analysis, and wrote the first draft of the manuscript. All authors contributed to evaluating subjects, manuscript revision, read, and approved the submitted version.

## Conflict of Interest

The authors declare that the research was conducted in the absence of any commercial or financial relationships that could be construed as a potential conflict of interest. The reviewer MD declared a past co-authorship with one of the authors JB to the handling editor.

## Publisher’s Note

All claims expressed in this article are solely those of the authors and do not necessarily represent those of their affiliated organizations, or those of the publisher, the editors and the reviewers. Any product that may be evaluated in this article, or claim that may be made by its manufacturer, is not guaranteed or endorsed by the publisher.

## Supplementary Material

Supplementary Figure 1 Diagram of study design. **(A)** Data analysis 1: rare, predicted-pathogenic variants were sought in 42 genes associated with pituitary gland development. **(B)** Data analysis 2: rare, predicted-pathogenic variants were sought in any gene in the genome (to capture undiscovered causes) that was inherited in a fashion that could explain the sporadic occurrence of the proband’s condition with a monogenic etiology (*de novo* mutation, autosomal recessive, or X-linked recessive). HP, congenital hypopituitarism; NFSS, non-familial short stature.

Supplementary Table 1 Genetic variants found in 42 known pituitary-associated genes: ARID1B, ARNT2, BMP2, BMP4, CHD7, CDON, FGF8, FGF10, FGF18, FGFR1, GATA2, GLI1, GLI2, GLI3, GLI4, GLI5, GLI6, GPR161, HESX1, IGSF1, LHX3, LHX4, NR5A1, OTX2, PAX6, PITX1, PITX2, POU1F1, PROP1, SHH, SIX1, SIX2, SIX3, SIX4, SIX5, SIX6, SOX1, SOX2, SOX3, TBX19, TGIF, WNT5A.
