# Supplementary figures and images for: Evidence That the Etiology of Congenital Hypopituitarism Has a Major Genetic Component but Is Infrequently Monogenic
Source: Front Genet. 2021 Aug 11;12:697549. doi: 10.3389/fgene.2021.697549 (PMC8386283; doi:10.3389/fgene.2021.697549)

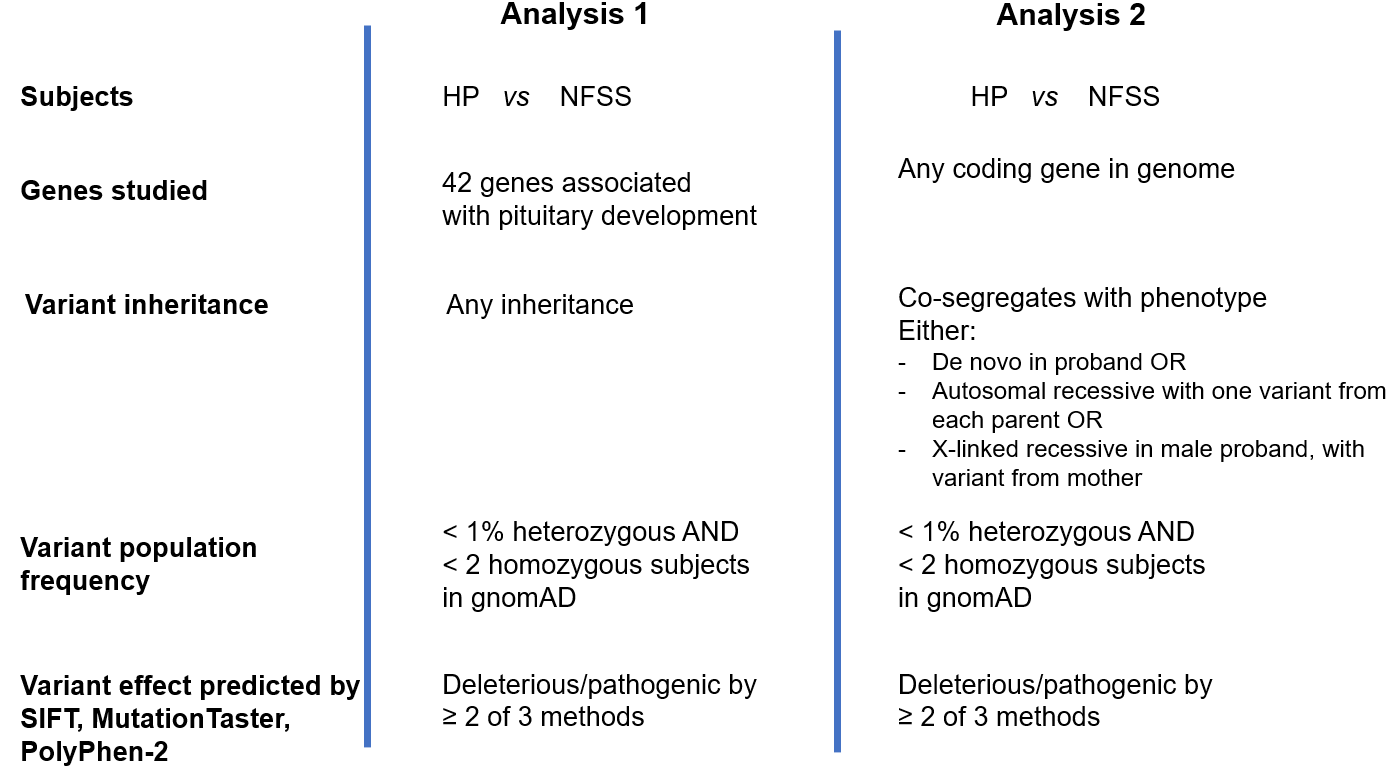

Supplement: Supplementary Figure 1 — Diagram of study design. (A) Data analysis 1: rare, predicted-pathogenic variants were sought in 42 genes associated with pituitary gland development. (B) Data analysis 2: rare, predicted-pathogenic variants were sought in any gene in the genome (to capture undiscovered causes) that was inherited in a fashion that could explain the sporadic occurrence of the proband’s condition with a monogenic etiology (de novo mutation, autosomal recessive, or X-linked recessive). HP, congenital hypopituitarism; NFSS, non-familial short stature. [file Image_1.tif]
