# Supplementary material for: Evidence That the Etiology of Congenital Hypopituitarism Has a Major Genetic Component but Is Infrequently Monogenic
Source: Front Genet. 2021 Aug 11;12:697549. doi: 10.3389/fgene.2021.697549 (PMC8386283; doi:10.3389/fgene.2021.697549)
Supplement: Supplementary Table 1 — Genetic variants found in 42 known pituitary-associated genes: ARID1B, ARNT2, BMP2, BMP4, CHD7, CDON, FGF8, FGF10, FGF18, FGFR1, GATA2, GLI1, GLI2, GLI3, GLI4, GLI5, GLI6, GPR161, HESX1, IGSF1, LHX3, LHX4, NR5A1, OTX2, PAX6, PITX1, PITX2, POU1F1, PROP1, SHH, SIX1, SIX2, SIX3, SIX4, SIX5, SIX6, SOX1, SOX2, SOX3, TBX19, TGIF, WNT5A. [file Table_1.DOCX]

**Supplemental Table 1. Genetic variants found in 42 known pituitary-associated genes; *ARID1B, ARNT2, BMP2, BMP4, CHD7, CDON, FGF8, FGF10, FGF18, FGFR1, GATA2, GLI1, GLI2, GLI3, GLI4, GLI5, GLI6, GPR161, HESX1, IGSF1, LHX3, LHX4, NR5A1, OTX2, PAX6, PITX1, PITX2, POU1F1, PROP1, SHH, SIX1, SIX2, SIX3, SIX4, SIX5, SIX6, SOX1, SOX2, SOX3, TBX19, TGIF, WNT5A***

|  | **Subjects with hypopituitarism** | |  | |
| --- | --- | --- | --- | --- |
| **Subject ID** | **Gene (variant, hg19)** | **SIFT/MutationTaster/Popyphen2** | **CADD score** | |
| HP subject 1 | *SIX5* (c.1681A>G:p.I561V,  NM_175875.4) | Tolerated/Polymorphism/Benign | 20.6 | |
| HP subject 2 | *CDON* (c.728G>A:p.C243Y,  NM_001243597.1)  *ARID1B* (c.2405C>T:p.S802L,  NM_001346813.1) | Deleterious/Disease causing/Probably damaging  Deleterious/NA/Probably damaging | 27.5  32 | |
| HP subject 3 | *SIX6* (c.385G>A:p.E129K,  NM_007374.2) | Deleterious/Disease causing/Possibly damaging | 31 | |
| HP subject 4 | None | . | . | |
| HP subject 5 | *SIX1* (c.746C>T:p.P249L,  NM_005982.3) | Deleterious/Disease causing/Benign | 24.6 | |
| HP subject 6 | *LHX4* (c.359G>A:p.C120Y,  NM_033343.4)  *CDON* (c.2663G>A:p.W888X,  NM_001243597.1)  *SIX5* (c.2146G>A:p.E716K,  NM_175875.4) | Deleterious/Disease causing/Probably damaging  Nonsense  Tolerate/Disease causing/Possibly damaging | 28  48  25.7 | |
| HP subject 7 | None | . | . | |
| HP subject 8 | *ARID1B* (c.4381C>A:p.P1461T,  NM_017519.2) | Tolerated/Disease causing/Possibly damaging | 28.2 | |
| HP subject 9 | *GLI1* (c.3307_3307del, NM_005269.2)  *SOX3* (c.720_740 del, NM_005634.2)  *CHD7* (c.2050_2058AAAGCAAAAGCAAAA) | frameshift  nonframeshift  nonframeshift | 35  16.3  21.1 | |
| HP subject 10 | *LHX4* (c.37G>A:p.V13I, NM_033343.4)  *SHH* (c.841G>A:p.A281T, NM_000193.2)  *GLI4* (c.565G>C:p.G189R, NM_138465.3) | Tolerated/Disease causing/Benign  Tolerated/Polymorphism/Benign  Deleterious/Polymorphism/Possibly damaging | 22.2  10.04  27.7 | |
| HP subject 11 | None | . | . | |
| HP subject 12 | *PAX6 (*c.*21_*22del NM_001258462.1) | Unknown pathogenicity | 0.3 | |
| HP subject 13 | *PAX6 (*c.*21_*22del NM_001258462.1)  *LHX3* (c.377 C>A, NM8138.6) | Unknown pathogenicity  Tolerated/Disease causing/Probably damaging | 0.3  22.1 | |
| Variants in gray did not meet the criteria to be considered potentially pathogenic candidate. | | | |  |
